# Mesenchymal stem cells/multipotent stromal cells (MSCs) are glycolytic and thus glucose is a limiting factor of in vitro models of MSC starvation

**DOI:** 10.1186/s13287-016-0436-7

**Published:** 2016-12-01

**Authors:** Austin Nuschke, Melanie Rodrigues, Albin W. Wells, Kyle Sylakowski, Alan Wells

**Affiliations:** 1 Department of Pathology, University of Pittsburgh School of Medicine, Scaife Hall, 3550 Terrace St, Pittsburgh, PA 15261 USA; 2 McGowan Institute for Regenerative Medicine, University of Pittsburgh, 450 Technology Drive, Pittsburgh, PA 15219 USA; 3 VA Pittsburgh Health System, University Drive A, Pittsburgh, PA 15261 USA; 4 Taylor Allderdice High School, 2409 Shady Ave, Pittsburgh, PA 15217 USA; 5 Present Address: Department of Plastic and Reconstructive Surgery, Stanford University, Palo Alto, CA USA

**Keywords:** Multipotent stem cells, Mesenchymal stem cells, Glucose metabolism, Nutrient starvation, Stem cell survival

## Abstract

**Background:**

Mesenchymal stem/multipotent stromal cells (MSCs) contribute to tissue repair but are challenged during wound healing when the blood supply is disrupted, thereby limiting nutrient delivery. Survival mechanisms against ‘starvation’ include autophagy, which we previously found to enhance differentiation efficiency. MSC response to models of in vitro nutrient deprivation are of great interest for improving MSC survival and therapeutic efficacy; however, the rate-limiting nutrients are unknown.

**Methods:**

MSC responses to culture nutrient and/or serum deprivations were assessed through light microscopy, cell survival, and measurements of metabolic levels. Glucose uptake was determined through conditioned media analyses over 3 days of culture. The Seahorse XF24 Flux analysis system was used to determine oxygen consumption and extracellular acidification for glycolytic metabolism. MSC autophagic response to these conditions was assessed via immunoblots for LC3-I and LC3-II, markers of autophagosome turnover.

**Results:**

We more closely examined limiting nutritional factors to MSC survival in vitro, finding that glucose is rapidly utilized/depleted whereas amino acids and other required nutrients were used sparingly. This finding concurred with metabolic analyses that showed a primarily glycolytic character to the MSCs at steady state. MSC autophagy, previously linked to MSC function through a unique accumulated autophagosome phenotype, also responded quickly to changes in glucose concentration, with drastic LC3-II changes within 24 h of glucose concentration shifts.

**Conclusions:**

Our results demonstrated a rapid uptake of glucose in MSC cultures that was due to a highly glycolytic phenotype for the cells; MSC starvation with serum or other nutrients appears to have a less notable effect on the cells. These findings highlight the importance of glucose and glucose metabolism on MSC function. The conditions and cellular responses outlined here may be essential in modeling MSC nutrient deprivation.

**Electronic supplementary material:**

The online version of this article (doi:10.1186/s13287-016-0436-7) contains supplementary material, which is available to authorized users.

## Background

Mesenchymal stem cells/multipotent stromal cells (MSCs) are key to tissue regeneration after injury, and attractive candidates for cell therapies due to a variety of paracrine benefits and capacities to differentiate [1, 2]. One major challenge faced by these cells that are present in all tissues is that wounding disrupts the blood supply that brings nutrients. A key response to cellular starvation is autophagy, which has been recently reported to occur in MSCs at the start of differentiation in a manner that enhances the efficiency [3, 4]. Thus, to contribute to repair, the MSCs must survive and subsequently differentiate or secrete beneficial factors in harsh environments.

We sought to determine what might trigger this process. Many investigators use an in vitro ‘starvation’ protocol to mimic the in vivo situation [5, 6]. They found that serum-free media induced changes in MSC phenotype but did not define the key nutrients. Here, we evaluated the key role of nutrients in MSC survival, focusing on modeling nutrient uptake and deprivation in vitro as a means of assessing MSC survival in implant sites. Briefly, we found rapid uptake of glucose in MSC cultures, coinciding with a glycolytic MSC phenotype that suggests a key role for glucose in implant sites or approaches to extending MSC lifespan. We also found that MSC autophagy, which we have previously found is a unique and important process in MSC function [4], responded rapidly to changes in glucose concentration. Interestingly, in a separate series of experiments, oxygen deprivation did not increase autophagy, and our calculations suggested that only in near anoxic conditions (<1%) would this be rate limiting; thus, we did not isolate this nutrient in these in vitro manipulations. Given the lack of change we found with other nutrient depletions, consistent with our calculations, our results suggest a key role for glucose in MSC function. Our results provide evidence for a glycolytic metabolism in MSCs, stressing the importance of nutrient/glucose supply in implant sites to extend MSC survival and clinical utility in cell therapies.

## Materials and methods

### Reagents

DMEM (10-014-CV) and α-MEM (15-012-CV) for MSC cultures were obtained from Corning/Mediatech (Manassass, VA, USA). In glucose experiments, phenol red-free DMEM (A14430-01) was obtained from Gibco/Thermo Fisher and α-MEM for primary cells (17-305-CV) was obtained from Corning. For cell culture preparations, fetal bovine serum (FBS) was obtained from Atlanta Biologicals (S11550H; Flowery Branch, GA, USA) for primary MSC cultures and Gemini Bio-Products (100-106; Sacramento, CA, USA) for immortalized MSC cultures. For the propidium iodide (PI) uptake experiments, propidium iodide from Thermo Fisher (P3566) was diluted in DMEM at 5.0 μg/mL. For immunoblotting, rabbit polyclonal LC3 antibody (NB100-2331) was obtained from Novus Biologicals (Littleton, CO, USA) and goat anti-rabbit IgG secondary antibody (A9169) was obtained from Sigma-Aldrich (St. Louis, MO, USA). Housekeeping gene anti-actin produced in rabbit (A2668) was obtained from Sigma-Aldrich. Protein ladder for all immunoblots was a Full Range Rainbow marker (RPN800E) from GE Life Sciences (Pittsburgh, PA, USA).

### MSC cell culture

Immortalized bone marrow-derived human MSCs were obtained as a kind gift from Dr. Junya Toguchida at Kyoto University. Passage 11 immortalized cells were expanded in DMEM supplemented with 10% FBS, 2 mM l-glutamine, 100 units/mL penicillin/streptomycin, 1 mM sodium pyruvate, and 1 μM non-essential amino acids. For glucose media analysis experiments, cultures were performed in phenol-red free DMEM with the same nutrient profile. Primary bone marrow-derived MSCs were obtained from the repository at the Prockop laboratory at Texas A&M; passage 3 MSCs were expanded in α-MEM supplemented with 16.5% FBS and 2 mM l-glutamine. For primary MSC experiments, repeats were performed with separate stocks at different time points. These ensured both technical and biological replicates and reproducibility.

For nutrient deprivation experiments, immortalized MSCs were seeded to near confluence and cultured in fully supplemented DMEM, complete DMEM without FBS, complete DMEM without l-glutamine, sodium pyruvate, and non-essential amino acids, or un-supplemented DMEM for up to 144 h. In a subset of experiments, propidium iodide at 5.0 μg/mL was directly added to the culture media to assess PI uptake. Cell detachment and PI fluorescence were assessed every 24 h for 144 h in total. For PI analysis, red PI fluorescence was quantitated using ImageJ, where all 10× images were converted to 8-bit format and the threshold was reduced to highlight the red punctae in culture, using the same thresholding for all analyzed images. Particle analysis in ImageJ was set to detect any fluorescent points with a radius greater than 100 pixels to exclude debris and background fluorescence. Percent area occupied by PI+ cells was calculated as an average for each nutrient deprivation.

### Glucose uptake

Immortalized MSCs were expanded at low (1000 cells/cm^2^ seeding density) and high (4000 cells/cm^2^ seeding density) confluence for 3 days in standard DMEM culture. Glucose uptake began with a change to phenol red-free DMEM, and subsequent collection of conditioned media was performed after 2, 4, 6, 8, 12, and 24 h in triplicate. Conditioned media was assessed in the College of American Pathologists certified clinical laboratories in the University of Pittsburgh Medical Center (UPMC, Pittsburgh, PA, USA) in accordance with governmental regulations, and average glucose concentrations were assessed over time to determine the rate of uptake and time to depletion. A replicate experiment was performed on primary MSCs seeded at 250 cells/cm^2^ and 1000 cells/cm^2^ to reflect comparable changes in density.

### MSC metabolism analysis

Energetics of the MSCs were determined using the Seahorse X24 Flux Analyzer (Seahorse Biosciences, Billerica, MA, USA) as previously published [7]. Briefly, a two-step seeding procedure was used to obtain a monolayer of cultured MSCs; 60,000 MSCs in 100 μL MSC media were grown per well of a Seahorse cartridge. The cells were allowed to attach for 4 h and then 150 μL MSC media was added to the cells, leaving four wells of the cartridge blank for temperature correction. Then 1 mL Seahorse Bioscience XF24 calibrant pH 7.4 was added to each well of a 24-well plate and placed below the cartridge. Cells were allowed to grow overnight at 37 °C under 5% CO_2_. The plates were checked for uniform spreading and cell confluency under the microscope 16 h after initial seeding. Growth media was aspirated, washed, and replaced with DMEM unbuffered. DMEM unbuffered was also added to the four temperature-correction wells. Basal oxygen consumption rate (OCR) and extracellular acidification rate (ECAR) were measured in the Seahorse XF24 Flux analyzer. Additional measurements were performed after injection of four compounds affecting bioenergetics: oligomycin (1 μM; Sigma-Aldrich), carbonyl cyanide 4-trifluoromethoxy-phenylhydrazone (FCCP) (300 nM; Sigma-Aldrich), 2-deoxyglucose (2-DG) (100 mM; Sigma-Aldrich), and rotenone (1 μM; Sigma-Aldrich). Upon completion of the Seahorse XF24 Flux analysis, cells were trypsinized, counted, and the results were normalized per 10^3^ cells.

Steady-state ATP levels were measured using the 2-step ATP Lite Luminescence Assay System (#6016941; Perkin Elmer, Waltham, MA, USA); 60,000 MSCs/well were grown overnight in 96-well black plates, followed by cell lysis using 50 μL of cell lysis solution and incubation for 5 min at 300 rpm. Thereafter, substrate solution (50 μL) was added, and the microplates were incubated for 5 min at 270 rpm. The plates were kept in the dark for 10 min, and luminescence was measured using a Biotek Synergy 2 plate reader (Winooski, VT, USA).

### Autophagy analyses

Immortalized MSCs at passage 11 were expanded in culture to 60% confluence in low (1 g/L) or high (4.5 g/L) glucose DMEM and subsequently shifted to the corresponding opposite glucose concentration for up to 72 h, with protein lysates collected at 1, 3, 6, 12, 24, 48, and 72 h for analysis. Lysates were probed for immunoblot using a myosin light chain 3 (LC3) antibody to examine LC3-I and LC3-II levels as a means to measure autophagosome turnover and accumulation.

## Results

### Nutrient depletion and starvation

**Fig. 1:**
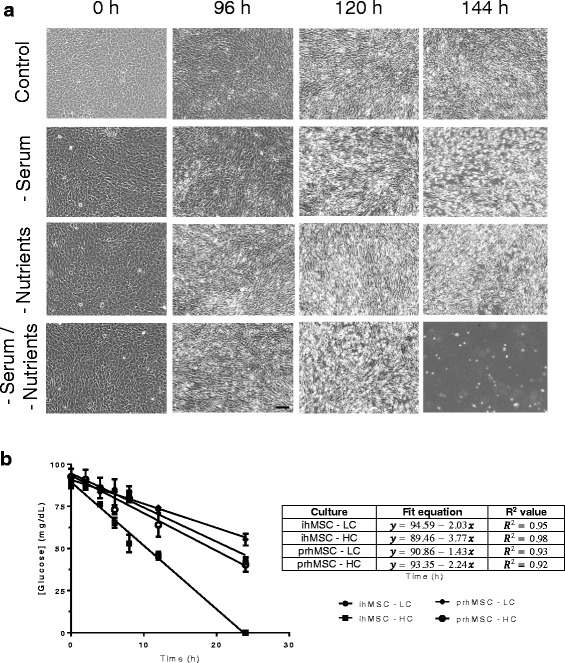
Glucose as a limiting nutrient in MSC cultures. Immortalized human mesenchymal stem cells/multipotent stromal cells (*ihMSC*) were seeded to confluence and monitored for detachment over 144 h, assessed every 24 h via 10× transmitted light microscopy of the live cells (**a**). Complete DMEM (*Control*), DMEM without serum (*-Serum*), DMEM without standard culture nutrient supplements (l-glutamine, non-essential amino acids, and sodium pyruvate; *-Nutrients*), and un-supplemented DMEM (*-Serum/-Nutrients*) were assessed as models of nutrient deprivation, with MSCs monitored until apparent apoptosis in culture. Cells did not exhibit a response to these deprivations until approximately 96 h. Immortalized and primary MSCs (*prhMSC*) were then seeded at varying confluence and allowed to proliferate at basal state for up to 48 h. Media from cultures were collected and assessed for glucose concentration to determine uptake rates and time to depletion (**b**). Experiments are averages over three experiments, *N* = 3. Images shown of MSC cultures are representative fields of cells in culture over three experiments. *HC*- high confluence, *LC*- low confluence

MSC response to available nutrients is a key component of efficacy in cell therapy. We sought to model MSC response to a variety of nutrient conditions, both as a natural depletion through normal cellular metabolism as well as in response to in vitro starvation. MSCs are cultured in vitro in a nutrient-rich environment consisting of fetal bovine serum and non-essential amino acids/sodium pyruvate/l-glutamine-supplemented media; as such we assessed starvations for these nutrients singularly and in concert over a period of a week in vitro. Serum deprivation did not produce an apparent apoptotic response in the immortalized human MSC (ihMSCs) until ~120 h of deprivation (Fig. 1a). Incorporation of propidium iodide indicative of cell membrane compromise demonstrated relatively comparable effects for nutrient deprivations across 96–144 h with little death prior to that; the un-supplemented media again presented the highest level of death (Additional file 1: Figure S1). Supplemental nutrient deprivation produced an even less dramatic effect over the same time scale. In combination, a total nutrient deprivation in vitro yielded an apoptotic phenotype beginning around 96 h.

This was quite unexpected, as the cells remained viable after 4 days without supplements. Thus, we queried the uptake of nutrients in the basal media. We had calculated that the media contained sufficient amino acids and glucose to not be depleted within two doublings; these calculations were based on published data for differentiated cells [8, 9]. These calculations were borne out by direct measurement in the case of amino acids (Additional file 2: Figure S2); the only amino acid to undergo significant loss within 3 days was glutamine, and this was due to hydrolysis (as also seen by the increase in glutamic acid, and synthesis of glycine and proline).

However, we were surprised that glucose underwent rapid depletion. We analyzed glucose depletion via conditioned media analysis for MSCs seeded at low (~20%) and high (80%) confluence over the course of 24 h to establish a rate of uptake in addition to a time to depletion for starvation modeling purposes. We found, strikingly, that glucose is rapidly depleted regardless of confluency in vitro, with immortalized MSCs at high confluence showing a total depletion of available glucose in standard DMEM culture media after 24 h (Fig. 1b). The uptake rate was remarkably linear over the course of the 24 h analyzed, and a regression analysis for the high confluence condition yielded a rate of depletion of 3.77 mg/dL/h. Immortalized MSCs seeded at low confluence demonstrated a lower rate of uptake as expected, with a linear rate of 2.03 mg/dL/h uptake and a time to depletion of approximately 46.5 h. As the high confluency condition was seeded at approximate four times the density of the low confluency condition, and the rate of uptake doubled on the same scale, it is apparent that glucose uptake scales with cell density at approximately half the change in cell density. In either case, available glucose was depleted rapidly in a linear fashion. Primary human bone marrow-derived MSCs showed comparable trends, with similar rates as the low-density immortalized MSCs. Confluency had less of an effect with the primary cells seeded in comparable conditions. These data presented glucose as a possible survival limit for MSCs.

### Glucose metabolism

The highly critical nature of glucose uptake by MSCs prompted us to investigate the metabolic nature of the cells given the unexpected high rate of glucose consumption from calculations based on oxidative phosphorylation. Glucose metabolism has been a somewhat controversial subject in MSC studies previously, with different studies suggesting an oxidative and glycolytic character for MSCs at a basal state [10, 11]. We subjected immortalized MSCs to a Seahorse analysis to determine oxygen consumption rates and extracellular acidification rates to characterize the glucose metabolism during this rapid uptake period.

This analysis provides an overview of glucose metabolism through oxygen consumption rate (OCR) and extracellular acidification rate (ECAR) output in response to four separate treatment points, where OCR is primarily an output of mitochondrial respiration and ECAR is more primarily a result of glycolytic behavior. Oligomycin treatment is used as an inhibitor of ATP synthase to assess ATP production levels, carbonyl cyanide-4-(trifluoromethoxy)phenylhydrazone (FCCP) treatment is used as a mitochondrial uncoupler to assess maximal respiration, 2-deoxy-d-glucose (2-DG) is used as a glucose analog to inhibit glycolysis, and rotenone treatment is used as a mitochondrial complex I uncoupler to assess non-mitochondrial respiration. A more detailed explanation of Seahorse analysis and interpretation can be found in Additional file 3: Figure S3.

**Fig. 2:**
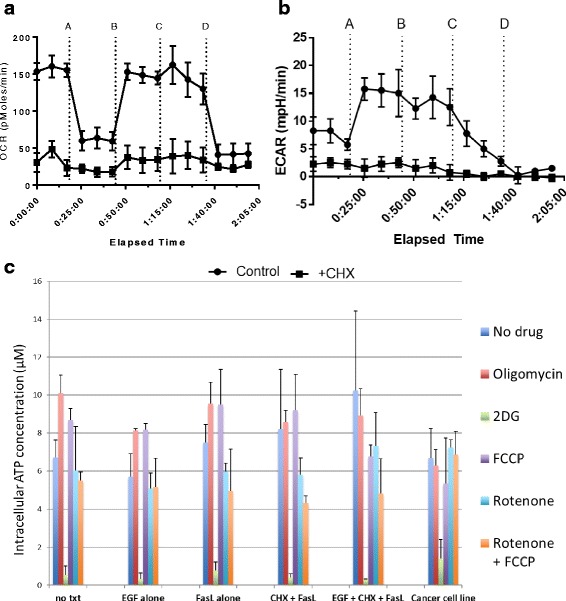
Glucose metabolism in MSC cultures. Immortalized MSCs in culture were subjected to Seahorse analyses to monitor glucose utilization via oxygen consumption rate (*OCR*) (**a**) as an assessment of oxidative phosphorylation and extracellular acidification (*ECAR*) (**b**) as an assessment of glycolysis (additions were 10 μM oligomycin at *A*, 3000 nM carbonyl cyanide-4-(trifluoromethoxy)phenylhydrazone (*FCCP*) at *B*, 1000 mM 2-deoxy-d-glucose (*2DG*) at *C*, and 10 μM rotenone at *D*; see Additional file 3: Figure S3 for a further description of interventions). Cycloheximide (*CHX*) treatments were included as a baseline non-respiratory control. Intracellular ATP in response to 2-h treatments was also assessed (**c**). Results showed repeatable trends over three separate studies. *N* = 4. *EGF-Epidermal Growth Factor*, *FasL-Fas Ligand*, *txt-Treatment﻿﻿*

MSCs sampled here demonstrated a relatively high extracellular acidification (ECAR) at steady state (7–8 mPH/min). ECAR in response to 2-DG was abolished, suggesting a high MSC dependence on glycolysis in vitro (Fig. 2b). Conversely, oxygen consumption rate (OCR) was largely unaffected by inhibitor treatments over time, and a lower basal OCR level persisted while ECAR depleted, suggesting a high dependence on glycolytic machinery (Fig. 2a). This was further confirmed by an analysis of intracellular ATP generation in response to comparable inhibitor treatments. ATP levels in response to 2 h of 2-DG treatment were essentially lowered to zero, while ATP levels persisted in response to oxidative phosphorylation inhibitors in the same MSC population (Fig. 2c). These results suggest a substantially glycolytic phenotype for MSCs at a basal state, in conjunction with a high dependence on glucose for normal metabolism and function.

### MSC autophagy

**Fig. 3:**
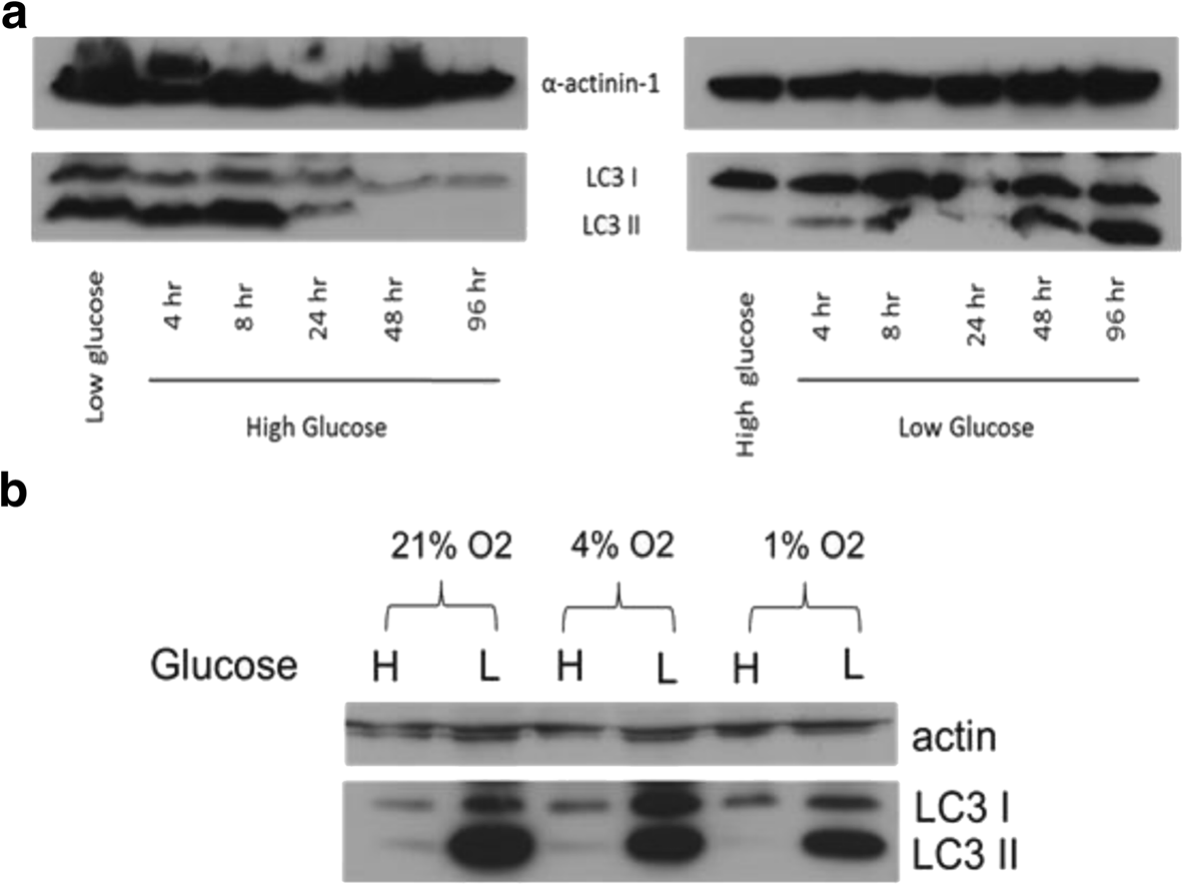
Autophagy responds rapidly to changing glucose conditions. Immortalized MSCs were cultured in physiologic (also called low in culture parlance; 1 g/L; 5.5 mM) or high (4.5 g/L; 25 mM) glucose media for 2 days and then changed to the corresponding opposite glucose concentration for up to 96 h. Myosin light chain 3 (*LC3*) levels were probed via immunoblot to assess autophagic response (**a**). The role of oxygen in the glucose response was also assessed by culturing the MSCs in a Biospherix hypoxic chamber at 4% and 1% oxygen in high and low glucose media for 4 days, followed by comparable LC3 blots (**b**). Shown are representative blots of three repeated studies. α-Actinin was used as a housekeeping control for all blots

We have previously found that MSC autophagy is intimately tied to normal MSC function in differentiation through a unique phenotype of accumulated autophagosomes in vitro [4]. This could explain how the cells survive the depletion of glucose, both in vitro and in vivo. Despite the lack of autophagic change in response to serum and supplemental nutrient deprivations (data not shown), we sought to establish the response of this key MSC process to glucose treatments due to the evident high dependence of MSCs on glucose for normal function. We cultured MSCs in both normal (1 g/L) and high (4.5 g/L) glucose media and subsequently changed the cultures to the corresponding opposite concentration for autophagy analysis over the course of 72 h. For these analyses we utilized the autophagy marker LC3-II. This marker is conjugated to autophagosome membranes from the cytosolic LC3-I form as autophagosomes form during the process of autophagy; as such, LC3-II turnover can be used to monitor autophagic turnover. We found moving the MSCs to a hyperglycemic condition induced a clearance in accumulated autophagosomes after approximately 24 h of culture, as evidenced by a rapid clearance of LC3-II levels in immunoblot analysis (Fig. 3a). Conversely, moving the MSCs from a hyperglycemic to normal glucose media re-induced the accumulation of the autophagosomes on a similar time scale of 24 h. In concert with the 24-h depletion of glucose seen in Fig. 1, these results support a high dependence of MSCs on normal glucose metabolism and availability for standard function in culture.

We additionally sought to assess the dependence of this autophagy switch on oxygen conditions, another potential stressor for MSCs upon implant, particularly if the MSCs were not glycolytic. We found oxygen did not affect the glucose phenotype, as MSC cultures for 48 h at both 4% and 1% oxygen did not change the autophagosome accumulation (or lack thereof) in both glucose conditions (Fig. 3b), further supporting the glycolytic nature of the cells.

## Discussion

MSC therapy remains a highly promising approach in tissue regeneration, immunomodulation, paracrine support, and other relevant areas, yet it is continually limited by the challenges MSCs face upon implant. Strategies to improve resistance to death stimuli are therefore of interest, as well as considerations for the challenges the implanted cells face. We have previously reported strategies to limit the induced death in these cells by engineering the matrix to present tethered EGFR ligand [12, 13]. However, this survival does not relate to the fate of such cells in the presence of low to minimal nutrients. Here, we have focused on nutrient deprivation/starvation models in vitro to examine both MSC reliance on nutrients as well as the related metabolism. Our models demonstrated a heavy reliance of MSCs on glucose, both through uptake and response to concentration as seen in the autophagic response. In addition, we found related evidence of a primarily glycolytic phenotype for the MSCs. We also found a relatively low response to other potential nutrient deprivations of interest, such as hypoxia or serum deprivation. The caveat of extrapolation to in vivo conditions is that the deprivations faced by stem cells are likely to be a simultaneous myriad, including loss of trophic factors (serum) and near anoxic conditions in addition to low glucose. The confounding influences are being studied in ongoing experiments that are beyond the scope of the present communication. However, to model the situation in vitro, the contributions were parsed in isolation, and glucose depletion was found to have an out-sized effect. These data outline glucose as a key consideration for MSC success in implant sites devoid of nutrient sources through available vasculature, whether it be a chronic skin wound or myocardial infarct site.

A key result from these studies is the role of glucose in MSC function, which was rapidly depleted by the cells in culture while metabolic analyses of the MSCs showed a relatively high glycolytic character. These results support recent reports that describe the metabolic flexibility of mesenchymal stem cells, with a high dependence on glycolysis and glucose metabolism [11, 14]. The metabolic flexibility reported by Mylotte et al. [11] may be key here, and the metabolic state of the cells both prior to and after implant for cell therapy or even differentiation in vitro seems to be key in determining the survival of the cells, activation of intracellular processes such as autophagy, and ultimately the outcomes from MSC use in translational work. This also expands on the role of glucose metabolism in stem cell function, which has recently shown a glycolytic character for hematopoietic stem cells and induced pluripotent stem cells in specific cell states or during reprogramming, respectively [15–17].

Beyond showing a heavy reliance on available glucose for normal function, our results show a striking lack of response by the MSCs to serum deprivations, as 30-min serum starvations are a typical design for MSC starvation or inducing autophagy in the cells. Western blots for LC3 demonstrated very little change in autophagy in the assayed MSCs until after 96 h of deprivation, suggesting the short serum deprivations used to induce starvation in these cells may be inadequate. This may be due to subtle differences between what amounts to a trophic factor deprivation in transient serum starvations, with resultant changes due to alterations of activation of cognate receptors, versus a true nutrient starvation of the cells; that is, a total depletion of available nutrients for uptake and subsequent changes in cellular processes. Given the role of serum starvations in many in vitro protocols, these data may prove important in at least establishing specific conditions for varying MSC populations, depending on the desired outcome for the cells. Our results also demonstrated a general lack of uptake for a variety of other nutrients, including supplemental amino acids, sodium pyruvate, and oxygen, all of which are key additives to standard MSC media and of interest in in vivo implant sites. MSC uptake of amino acids proved to be very slow in the immortalized cells assayed here, with only l-glutamine showing substantial loss, and that being due to hydrolysis rather than cell-mediated processes [18]. Starvation of these nutrients also took several days to show a demonstrable effect on the MSCs in culture, as seen in Fig. 1. Calculations of oxygen diffusion and uptake also demonstrated a low reliance of MSCs on available oxygen, despite the multitude of reports showing the effects of hypoxia on MSC function [19, 20]. This suggests a potential role of oxygen in regulating MSC function but not in acting as a limiting reagent for MSC survival necessarily, and is an important consideration in applying MSCs to hypoxic implant sites in vivo.

Results from these studies also build on the importance of autophagy in normal MSC function. Several studies from our group and others have outlined the key role of autophagy in MSC stress responses, including differentiation, death resistance, and others [3, 4, 21]. As implanted MSCs are immediately subject to a lack of available nutrients, the rapid autophagic response seen in MSCs exposed to changing glucose concentrations further outlines the crucial nature of basal autophagy and autophagosome cycling in the MSC response to changing conditions and stressors. Taking advantage of the key role of autophagy remains a potentially useful strategy in priming the MSCs for optimal function both in vitro and in vivo moving forward.

## Conclusions

In summary, our results demonstrate a key role of available glucose and related metabolism in survival and normal function of mesenchymal stem cells, especially in comparison to other common nutrients of interest in culture or in implant sites. The optimal function of MSCs in culture and survival/function upon implantation for cell therapy remains critical in the successful use of MSCs. We have here established the importance of glucose and glucose metabolism in MSC function, while also showing at least the potential for variability among MSC populations in dependence on serum and other culture nutrients. The role of nutrient availability and MSC response as such is an important and often overlooked consideration for MSC stress response and warrants further investigation and optimization in relation to MSC cell therapies and in vitro protocols.

## Additional files

Additional file 1: Figure S1. PI uptake during nutrient deprivations. To validate the cell death indicators noted in Fig. 1, immortalized MSCs were cultured for up to 144 h with 5.0 μg/mL propidium iodide in the culture media to allow for PI uptake and punctae formation as the cells were starved. Red PI fluorescence was quantified via ImageJ threshold analysis at the time points correlating with results in Fig. 1. Percent area calculated from particle analysis via thresholding was averaged across two fields per time point. (PDF 181 kb)
Additional file 2: Figure S2. MSC amino acid and oxygen uptake. Immortalized MSCs were seeded in culture and grown to 70% confluence and then allowed to proliferate for up to 72 h in control and serum-free media. Media was collected from samples in triplicate at 24, 48, and 72 h and a global nutrient assessment was performed for concentrations of amino acids and other relevant nutrients. A selection of nutrients with appreciable concentrations at the time points assessed is shown in (A). Three separate experiments are represented in the data with less than a 5% deviation in all analytes across the experiments. Oxygen was also investigated as a nutrient of interest for the MSCs. Theoretical oxygen uptake was calculated as a function of number of MSCs in our cultures and determined to have little effect on the time scale assessed in this study (B). Only in extreme conditions did the flux delivery of oxygen fall below the approximate glycolytic uptake of 25 pM/10^4^ cells/min (red box in table). (PDF 226 kb)
Additional file 3: Figure S3. Theoretical interpretation of Seahorse metabolism output. Data on oxygen consumption rate (OCR) and extracellular acidification rate (ECAR) adapted from the Seahorse Bio manual are plotted and outlined to highlight treatment rationale and subsequent interpretation. For OCR (left), basal mitochondrial respiration (A; blue) is disrupted with oligomycin treatment to inhibit ATP synthase and demonstrate ATP consumption (B; red), followed by FCCP treatment to uncouple the mitochondria and demonstrate maximal respiratory capacity (C; green). Rotenone treatment to completely inhibit complex I is then used to reduce OCR to non-mitochondrial respiration only (D; yellow). For ECAR (right), a preliminary dose of glucose is used to stimulate extracellular acidification through glycolysis (A; blue), followed by oligomycin treatment to again inhibit ATP synthase and yield maximal glycolytic capacity (B; green). 2-DG is finally used as a glucose analog to competitively inhibit glycolysis and return ECAR to non-glycolytic acidification levels (C; yellow). In our studies, both OCR and ECAR were assessed simultaneously through four treatment points using oligomycin, FCCP, 2-DG, and rotenone in sequence (see Fig. 2). Maximal respiratory and/or glycolytic capacities were assessed using the same rationale following FCCP or oligomycin treatment, respectively. (PDF 119 kb)

## Abbreviations

ECAR: Extracellular acidification rate
FBS: Fetal bovine serum
LC3: Myosin light chain 3
MSC: Mesenchymal stem cell/multipotent stromal cell
OCR: Oxygen consumption rate
PI: Propidium iodide
