# Supplementary figures and images for: Mesenchymal stem cells/multipotent stromal cells (MSCs) are glycolytic and thus glucose is a limiting factor of in vitro models of MSC starvation
Source: Stem Cell Res Ther. 2016 Dec 1;7:179. doi: 10.1186/s13287-016-0436-7 (PMC5134064; doi:10.1186/s13287-016-0436-7)

Fig S1

0 h

96 h

120 h

144 h

Control

- Serum

- Nutrients

- Serum /  
- Nutrients

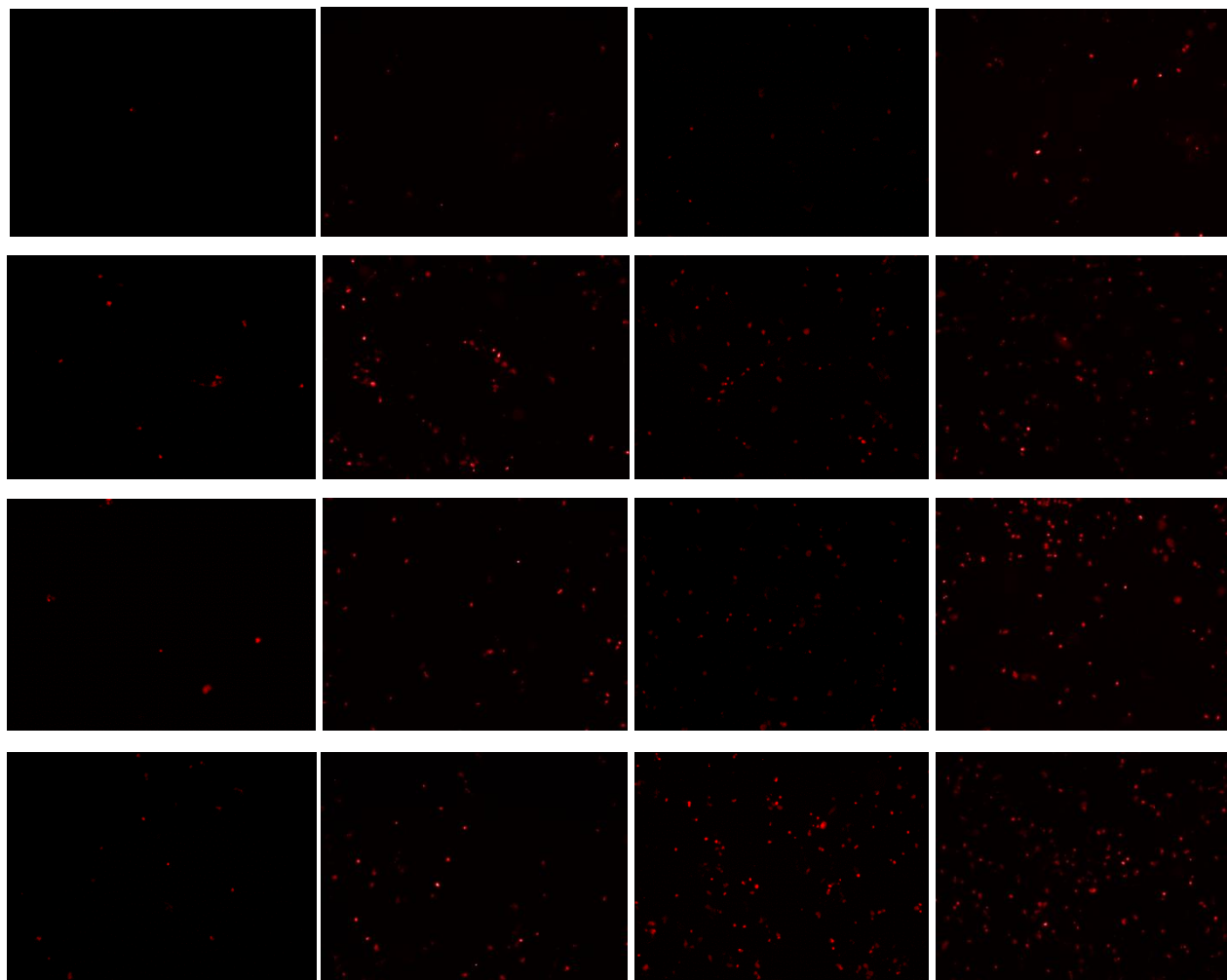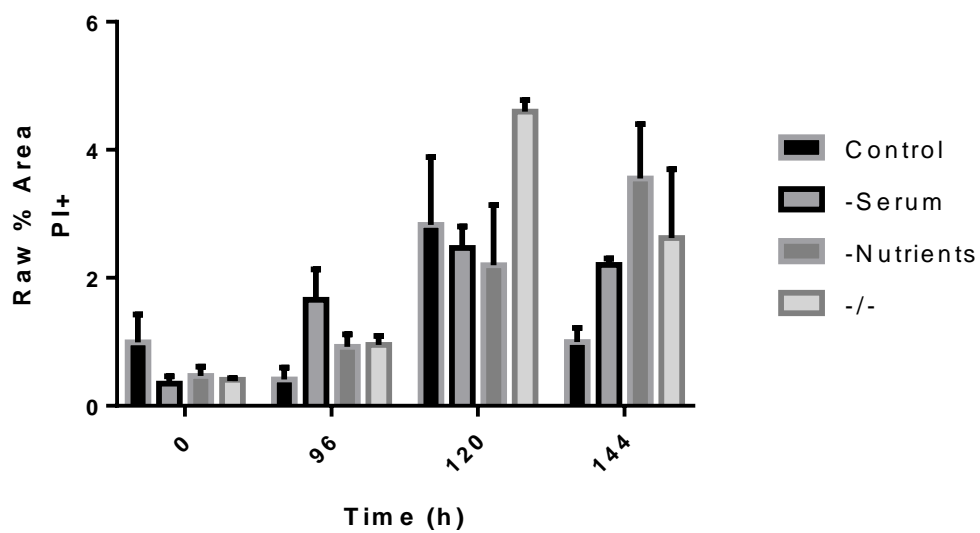

Supplement: Additional file 1: Figure S1. — PI uptake during nutrient deprivations. To validate the cell death indicators noted in Fig. 1, immortalized MSCs were cultured for up to 144 h with 5.0 μg/mL propidium iodide in the culture media to allow for PI uptake and punctae formation as the cells were starved. Red PI fluorescence was quantified via ImageJ threshold analysis at the time points correlating with results in Fig. 1. Percent area calculated from particle analysis via thresholding was averaged across two fields per time point. (PDF 181 kb) [file 13287_2016_436_MOESM1_ESM.pdf]

Fig S3

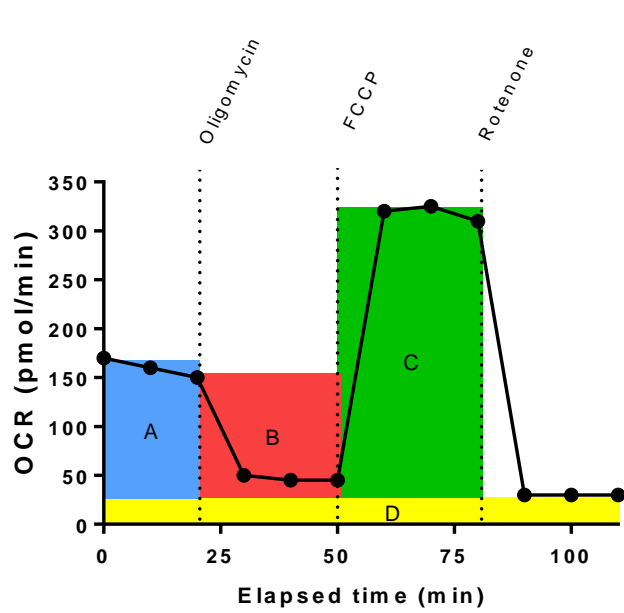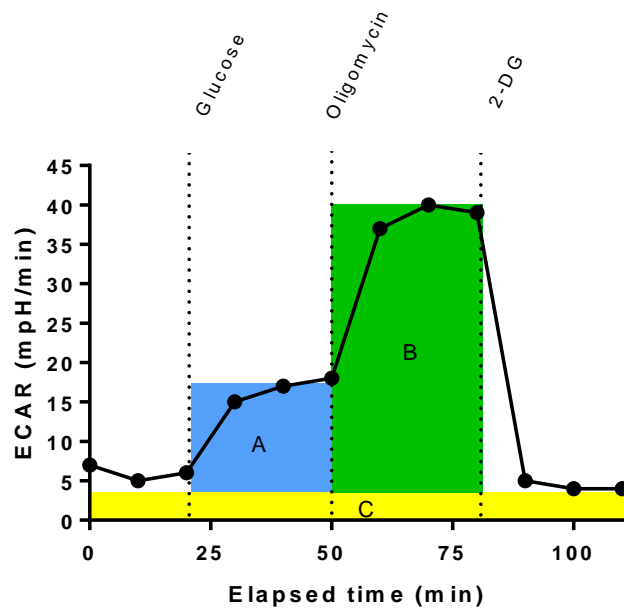

Supplement: Additional file 3: Figure S3. — Theoretical interpretation of Seahorse metabolism output. Data on oxygen consumption rate (OCR) and extracellular acidification rate (ECAR) adapted from the Seahorse Bio manual are plotted and outlined to highlight treatment rationale and subsequent interpretation. For OCR (left), basal mitochondrial respiration (A; blue) is disrupted with oligomycin treatment to inhibit ATP synthase and demonstrate ATP consumption (B; red), followed by FCCP treatment to uncouple the mitochondria and demonstrate maximal respiratory capacity (C; green). Rotenone treatment to completely inhibit complex I is then used to reduce OCR to non-mitochondrial respiration only (D; yellow). For ECAR (right), a preliminary dose of glucose is used to stimulate extracellular acidification through glycolysis (A; blue), followed by oligomycin treatment to again inhibit ATP synthase and yield maximal glycolytic capacity (B; green). 2-DG is finally used as a glucose analog to competitively inhibit glycolysis and return ECAR to non-glycolytic acidification levels (C; yellow). In our studies, both OCR and ECAR were assessed simultaneously through four treatment points using oligomycin, FCCP, 2-DG, and rotenone in sequence (see Fig. 2). Maximal respiratory and/or glycolytic capacities were assessed using the same rationale following FCCP or oligomycin treatment, respectively. (PDF 119 kb) [file 13287_2016_436_MOESM3_ESM.pdf]
