# Supplementary material for: Mesenchymal stem cells/multipotent stromal cells (MSCs) are glycolytic and thus glucose is a limiting factor of in vitro models of MSC starvation
Source: Stem Cell Res Ther. 2016 Dec 1;7:179. doi: 10.1186/s13287-016-0436-7 (PMC5134064; doi:10.1186/s13287-016-0436-7)

Fig S2

A)

	Concentration (uMol/dL)								
	Control			-Serum			-Nutrients		
**Nutrient**	**24 hr**	**48 hr**	**72 hr**	**24 hr**	**48 hr**	**72 hr**	**24 hr**	**48 hr**	**72 hr**
Glutamine	206	91	15	228	156	110	74	nd	nd
Proline	15	29	38	15	32	44	12	21	28
Glycine	60	81	92	60	83	96	50	65	74
Alanine	93	124	121	81	114	119	51	57	33
Leucine	50	54	53	55	62	66	55	53	47
Tyrosine	31	30	31	35	34	35	35	35	34
Lysine	60	60	60	69	77	81	69	76	81
Histidine	15	15	14	17	17	18	17	17	18
Aspartic Acid	9	6	4	8	5	3	8	5	3

B)

Oxygen flux (pM/10^4 cells/min)			
**height of media**	**20% oxygen**	**5% oxygen**	**1% oxygen**
**0.1cm**	1800	450	90
**0.2cm**	900	225	45
**0.5cm**	360	90	18

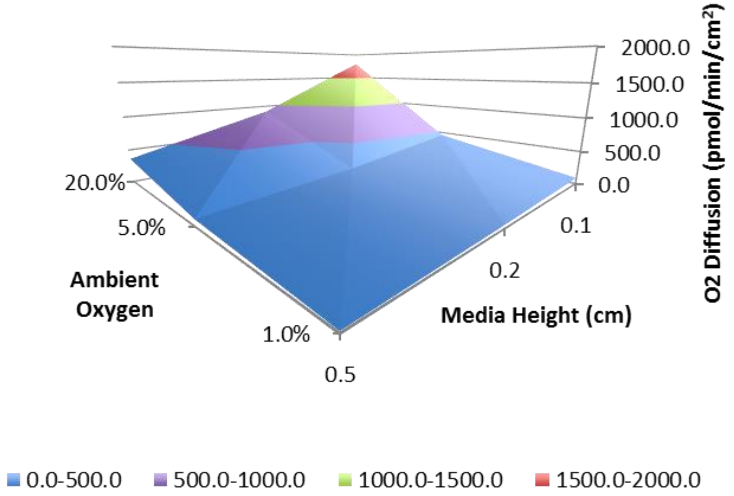

Supplement: Additional file 2: Figure S2. — MSC amino acid and oxygen uptake. Immortalized MSCs were seeded in culture and grown to 70% confluence and then allowed to proliferate for up to 72 h in control and serum-free media. Media was collected from samples in triplicate at 24, 48, and 72 h and a global nutrient assessment was performed for concentrations of amino acids and other relevant nutrients. A selection of nutrients with appreciable concentrations at the time points assessed is shown in (A). Three separate experiments are represented in the data with less than a 5% deviation in all analytes across the experiments. Oxygen was also investigated as a nutrient of interest for the MSCs. Theoretical oxygen uptake was calculated as a function of number of MSCs in our cultures and determined to have little effect on the time scale assessed in this study (B). Only in extreme conditions did the flux delivery of oxygen fall below the approximate glycolytic uptake of 25 pM/104 cells/min (red box in table). (PDF 226 kb) [file 13287_2016_436_MOESM2_ESM.pdf]
